# Yield, Phytonutritional and Essential Mineral Element Profiles of Selected Aromatic Herbs: A Comparative Study of Hydroponics, Soilless and In-Soil Production Systems

**DOI:** 10.3390/plants14142179

**Published:** 2025-07-14

**Authors:** Beverly M. Mampholo, Mariette Truter, Martin M. Maboko

**Affiliations:** 1 Agricultural Research Council, Vegetable, Industrial and Medicinal Plants, Roodeplaat, Pretoria 0001, South Africa; 2 Department of Crop Sciences, Tshwane University of Technology, Pretoria 0001, South Africa; mabokom@tut.ac.za; 3 Department of Agriculture and Animal Health, University of South Africa, Roodepoort 1709, South Africa

**Keywords:** aromatic herbs, nutritional quality, growing environments

## Abstract

Increased market demand for plant herbs has prompted growers to ensure a continuous and assured supply of superior nutritional quality over the years. Apart from the nutritional value, culinary herbs contain phytochemical benefits that can improve human health. However, a significant amount of research has focused on enhancing yield, frequently overlooking the impact of production practices on the antioxidant and phytonutritional content of the produce. Thus, the study aimed to evaluate the yield, phytonutrients, and essential mineral profiling in selected aromatic herbs and their intricate role in nutritional quality when grown under different production systems. Five selected aromatic herbs (coriander, rocket, fennel, basil, and moss-curled parsley) were evaluated at harvest when grown under three production systems: in a gravel-film technique (GFT) hydroponic system and in soil, both under the 40% white shade-net structure, as well as in a soilless medium using sawdust under a non-temperature-controlled plastic tunnel (NTC). The phytonutritional quality properties (total phenolic, flavonoids, β-carotene-linoleic acid, and condensed tannins contents) as well as 1,1-diphenyl-2-picrylhydrazyl (DPPH) were assessed using spectrophotometry, while vitamin C and β-carotene were analyzed using HPLC-PDA, and leaf mineral content was evaluated using ICP-OES (Inductively Coupled Plasma Optical Emission Spectrometry). The results show that the health benefits vary greatly owing to the particular culinary herb. The fresh leaf mass (yield) of coriander, parsley, and rocket was not significantly affected by the production system, whereas basil was high in soil cultivation, followed by GFT. Fennel had a high yield in the GFT system compared to in-soil and in-soilless cultivation. The highest levels of vitamin C were found in basil leaves grown in GFT and in soil compared to the soilless medium. The amount of total phenolic and flavonoid compounds, β-carotene, β-carotene-linoleic acid, and DPPH, were considerably high in soil cultivation, except on condensed tannins compared to the GFT and soilless medium, which could be a result of Photosynthetic Active Radiation (PAR) values (683 μmol/m^2^/s) and not favoring the accumulation of tannins. Overall, the mineral content was greatly influenced by the production system. Leaf calcium and magnesium contents were highly accumulated in rockets grown in the soilless medium and the GFT hydroponic system. The results have highlighted that growing environmental conditions significantly impact the accumulation of health-promoting phytonutrients in aromatic herbs. Some have positive ramifications, while others have negative ramifications. As a result, growers should prioritize in-soil production systems over GFT (under the shade-net) and soilless cultivation (under NTC) to produce aromatic herbs to improve the functional benefits and customer health.

## 1. Introduction

Aromatic herbs are herbaceous (leafy) plants that serve multifarious roles, as phytomedicine and condiments to enhance the flavor in culinary due to their phytonutritional properties [1] and broad market prospects. Besides adding flavor and aroma, aromatic herbs are consumed in modest quantities, providing numerous bioactive compounds that offer positive health benefits. Culinary herbs give dishes a delicacy, and they are primarily used in several forms, as dry or fresh herbs and as essential oils to fortify food product preservatives by delaying the onset of rancid development, thus improving overall food quality [2,3]. Over the last decade, there has been a major resurgence in the understanding cultivation of culinary herbs due to their distinctive variety of dietary phenolic compounds and their peculiar antioxidant properties in these plants. However, variability in phytonutritional concentrations in horticulture is frequently influenced by factors such as genetic factors (variety), production practices (fertilization, water and mineral nutrient availability, soil and crop management), and changing environments during plant growth and development as well as during processing and storage, leaving an indelible impact on crop quality [3,4]. Notably, the primary dietary phenolic antioxidant compounds that occur naturally in plants are simple phenolic acids, flavonoids, coumarins, stilbenes, hydrolysable, lignans and lignins, and condensed tannins produced through shikimic acid by the phenylpropanoid pathway, which play important roles in pabulum and medicine [5,6].

Various plant species produce phenylpropanoid compounds in response to photoinhibition and nutritional stressors such as nitrogen, phosphorus, potassium, sulfur, magnesium, boron, and iron deficiency. The structural diversity of phenolic compounds comprises an aromatic ring, bearing one or more hydroxyl groups. These compounds range from simple phenolic molecules to complex, highly polymerized compounds [6]. These substances contain diverse molecules with powerful antioxidant, anti-aging, anti-cancer, anti-asthmatic, and anti-inflammatory agents [6]. Antioxidant compounds in herbs are effective diuretics used in managing health disorders such as obesity. This severe global epidemic can result in numerous illness complications, including type 2 diabetes mellitus, heart disease, stroke, and some types of cancer in humans [7,8]. Herbal produce such as culinary herbs are used as medicinal due to their role in retarding the emergence of a wide range of reactive oxygen species (ROS), reactive nitrogen species (RNS), and chlorine species due to their phenolic antioxidant properties. Additionally, all these species are very dangerous to overall well-being and are implicit in various diseases, caused by such species such as superoxide, hydroxyl, and peroxyl radicals, as well as peroxynitrous acid and hypochlorous acid caused by lipid oxidation; thus, rapid donation of a hydrogen atom to lipid radicals by these phenolic antioxidants impede lipid oxidation [9,10].

Several mineral elements, particularly iron, potassium, and phosphorus, also contribute to glycemic management against health disorders [11]. Horticultural crop production is increasingly oriented toward high-quality products containing a variety of phytonutritinal compounds such as vitamins, flavonoids, antioxidants, and minerals. In light of the world’s expanding population, consumers are becoming more knowledgeable about nutrition because of trendy sedentary lifestyles, demanding culinary herbs produce with superior nutritional quality [12]. The production of herbal vegetables with high phytonutritional constituents and high-quality market sales is crucial for growers and processors to meet consumers’ eating aspirations while maintaining their health, and if quality is not met, the price of the produce becomes affected, as well as consumer acceptance [13]. For this study, it is critical to investigate the dynamics of various growing environmental conditions on the accumulation of these phytonutritional compounds and yield in aromatic herbs [14]. The study aims to assist culinary herb growers in understanding the impact of different production systems on yield, nutritional quantity, and phytonutritional accumulation.

## 2. Results and Discussion

### 2.1. Phytonutrional Profiling of Selected Herbs Grown Under Different Environmental Conditions

#### 2.1.1. Vitamin C Content

Vitamin C is the most abundant water-soluble antioxidant with pleiotropic functions capable of donating electrons and protecting host cells from the actions of ROS, such as superoxide anion, hydrogen peroxide, hydroxyl radicals, and singlet oxygen released by phagocytes [15]. Phagocytes have a unique transport system that allows the oxidized form of vitamin C (dehydroascorbic acid) to enter the cell and be converted into the reduced form of vitamin C [16]. Vitamin C plays an essential role in collagen formation, which is essential for preserving the structural integrity of the skin, connective tissues, and blood vessels. It is also an essential micronutrient that aids in bone formation, wound healing, and the maintenance of healthy gums [17]. Vitamin C promotes optimal acidic conditions for iron absorption, particularly in the stomach and intestines, and also functions as a chelating agent of ferric iron, keeping it stable and soluble even at higher pH levels [17,18]. A deficiency of vitamin C causes scurvy, a potentially lethal condition. Scurvy patients are particularly vulnerable to potentially fatal infections such as pneumonia and respiratory infections. The accumulation of phytochemicals in aromatic herbs depends on the species. Table 1 shows the significant effect of production systems on the vitamin C content of the herbs. Basil leaves produced in soil were significantly higher (*p* < 0.01) in vitamin C content (18.6 mg g^−1^), and this was over double the amount of that grown in the soilless medium. The increased vitamin C in basil grown in soil may be the result of plants being exposed to oxidative stress. Compared to a hydroponic growing environment, plant growth is optimized and reduces plant oxidative damage from fluctuations in the environment. Meanwhile, the basil grown in the GFT hydroponic system had the second most abundant vitamin C content, which is 1.3 times lower than the basil grown in soil but 1.7 times higher than the basil grown in soilless medium. The results show that the accumulation of vitamin C varied in aromatic herbs between growing environments because of different climatic conditions (PAR, humidity, fertilization, and temperature). PAR (light quantity) is an important environmental signal in regulating vitamin C levels, notably in leafy greens. Vitamin C is a byproduct of the conversion of sugars produced during photosynthesis; increasing sunlight stimulates photosynthetic processes and provides more starting material to produce ascorbic acid [19]. The vitamin C content is controlled by the amount and intensity of light during the growth season, but it is also a cultivar-dependent feature. The increased PAR (683 μmol/m^2^/s) values in shade-net production than NTC production could explain the high vitamin C accumulation in the tested herbs. Rocket grown in soil had the second highest in vitamin C content after basil. It was also high in the soilless medium compared to fennel, coriander, and parsley, which performed similarly in all production systems except for coriander in the soilless medium (Table 1). A higher light intensity, temperature, and B/R ratio increased the vitamin C content in basil leaves by promoting the production of photo-assimilate substrates (hexose and D-glucose sugars as precursors for vitamin C accumulation) [20]. Although the PAR and B/R ratios were lower in the soilless medium (under NTC), the ascorbate content of the leaves may have been altered by a range of environmental factors, including air temperatures [20,21]. Some research found that the increased vitamin C content in plants is due to the activity of enzymes related to this vitamin, which is associated with stress tolerance to unfavourable environmental conditions [21].

#### 2.1.2. β-Carotene Content

β-carotene, known as a key member of the carotenoid family, is recognized as one of the most potent antioxidants responsible for the distinctive colors (orange, yellow, and dark green) of various fruits and vegetables [22]. Fruits and vegetables rich in β-carotene include carrots, pumpkins, orange-fleshed sweet potatoes, tomatoes, beets, broccoli, mango, and leafy greens. This compound is a precursor to carotenoid vitamin A (retinol, which is found in the human diet) [23]. The health benefits of β-carotene are attributed to its given biological properties as antioxidants quench ROS, as provitamin A compounds that activate retinol-mediated pathways, and as electrophiles that boost endogenous antoxidant systems, by hampering inflammation-related processes mediated by the nuclear factor κ-light-chain-enhancer of the activated B cell (NF-κB) pathway and by directly binding nuclear receptors (NRs) and other transcription factors in target cells [24]. Young pre-school children and pregnant women are reported to be the most vulnerable groups for vitamin A deficiency in developing countries [25].

In general, the selected herbs demonstrated a significantly high β-carotene content. Plants have specialized photoreceptors to perceive light radiation to assimilate carbon by photosynthesis for their energy requirements of different wavelengths. Most aromatic herbs grown under NTC conditions (418 mol/m^2^/s PAR) showed similar growing trends in β-carotene as those grown under white shade-net (683 mol/m^2^/s PAR). Basil had the highest β-carotene when grown in soil compared to other herbs grown in different production systems. Generally, most herbs grown in GFT hydroponic systems, followed by in-soil production, had high beta-carotene, with the least recorded in the soilless medium. Table 1 shows that the herbs grown in soil and GFT under the shade-net had significantly higher β-carotene content due to high PAR (683 mol/m^2^/s), with basil demonstrating the highest content (39.9 mg/100 g) compared to other varieties, with parsley, rocket, coriander, and fennel having comparable content (31.5–36.7 mg/100 g). The high β-carotene content observed under GFT and shade-net conditions could also be attributed to the sun’s UV radiation index produced under the 40% shade-net compared to the NTC environment. UV radiation activates enzymes such as phenylalanine ammonium-lyase (PAL) and chalcone synthase (CHS) [26], which are both important enzymes in the phenylpropanoid pathway and are responsible for the synthesis of phenolic compounds. PAL is the primary enzyme in phenolic acid accumulation and biosynthesis [27,28]. Phenolic acids, such as chlorogenic acid and others, are UV-B absorbing compounds and may serve as UV-B receptors in plants. Light promotes the rapid synthesis of carotenoids in etiolated plants grown in low light previously. Being components of the photosynthetic system, the reduced light content may be a contributing factor to the lower content of β-carotene in herbs produced in tunnels. The amount of β-carotene in crops can be considerably influenced by other environmental elements, including temperature, growing medium, and fertilization. Plants grown in full sunlight, on the other hand, may experience photoinhibition since they require balanced light absorption. This could result in low amounts of photosynthetic pigments like carotenoids and total chlorophyll [29,30].

#### 2.1.3. Total Phenolic Content

The phenols are found in a wide variety of fruits, vegetables, grains, herbs, and spices and are classified based on their chemical structures and functional properties. In plant-microbe symbioses, phenolic compounds play an important role as signaling molecules [31]. They also function as antioxidants, structural polymers (lignin), attractants (flavonoids and carotenoids), UV screens (flavonoids), signal compounds (salicylic acid and flavonoids), and defense response chemicals (tannins and phytoalexins). These compounds serve as the body’s primary free-radical terminators, preferentially for hydroxyl and peroxyl radicals, preventing the unfavorable reactivity of reactive oxygen/nitrogen species produced by metabolic processes [32]. Plants use phenolic compounds for a variety of functions during growth, including food intake, enzyme activity, photosynthesis, and protein synthesis. In humans, phenolic compounds play an important role in defense responses such as anti-aging, anti-inflammatory, antioxidant, and anti-proliferative properties [33]. As a result, eating plant foods with a high antioxidant compound content can help to reduce the occurrence of chronic diseases by managing oxidative stress. Herbs and phenolic compounds add a distinct taste and flavor to dishes, making them incomparably delectable. The phenolic content of the herbs under study ranges from 3.9 mg GAE/g DW for parsley to 25.6 mg GAE/g DW for basil. The intensity of oxidative stress caused by environmental factors influences the production of phenolic compounds in plants [34]. A high phenol content was found in basil grown in soil production (25.6 mg GAE/g DW), followed by the GFT hydroponic system (17.1 mg GAE/g DW) and lastly in the soilless medium (13.2 mg GAE/g DW) compared to other herbs (3.9 to 9.1 mg GAE/g DW). Parsley tended to have a low phenol content when grown in GFT (3.9 mg GAE/g DW) and in the soilless medium (5.9 mg GAE/g DW). Generally, soil production showed increased phenol content amongst the herbs compared to other production systems (GFT and the soilless medium). It is expected that soil-grown produce will possess a significant amount of phenolic activity due to high light intensity. Therefore, a variety of limitations, including unsuitable environmental conditions and improper root access to water and nutrients, may be responsible for the increase in phenolic levels in soil cultivation as compared to hydroponic cultivation [33]. Based on this experiment, different production systems influenced the accumulation of total phenolic compounds in selected herbs grown under different environmental conditions.

#### 2.1.4. Total Flavonoid Content

Flavonoids perform a variety of important biological functions in plants. Flavonoids protect plants from biotic and abiotic stressors, act as a unique UV filter, and serve as a signal molecule, phytoalexins, detoxifying agents, spore germination stimulants, UV-filters, pollinator attractants, and allelochemical agents [35]. Flavonoids are found naturally in plants as aglycones, with sugars and hydroxyl groups making them water soluble and methyl groups and isopentyl units making flavonoids lipophilic [36]. Many factors influence flavonoid biosynthesis in plants, including meteorological conditions (light, humidity, temperature, altitude, soil type, and water), microbial interactions, and nutritional status [37]. As a result, interest in phenolic compounds derived from vegetables and their nutritional effects is growing. The variability in total flavonoid content is determined by the pigmentation pattern of the leaves [38]. The flavonoid content varied greatly between types and samples collected under different environmental conditions in the study, ranging from 3.0 to 6.9 mg CAE/100 g of DW (Table 1). The total flavonoid content was obtained from basil grown in soil cultivation (6.9 mg CE/g DW) while other herbs from production systems treatments performed similarly (4–5.5 mg CE/g DW) except fennel, which was the lowest when grown in soil (3.4 mg CE/g DW) and soilless medium 3.0 mg CE/g DW).

The basil planted in soil under a shade-net contained the most flavonoids (6.9 mg/100 g), and fennel produced in soilless medium (3.0 mg/100 g) under NTC yielded the lowest flavonoid content. The accumulation of β-carotene in different production systems for aromatic herbs is attributed to differences in plant growth conditions, such as water supply, nutritional status, temperature, relative humidity, and light intensity. High PAR (683 μmol/m^2^/s) has also been identified as a key regulator of flavonoid accumulation; the content of flavonoids in plants grown in soil under the shade-net and GFT have been induced by light.

The flavonoid content of the leaves is a major contributor to antioxidant activity, which protects the human body from oxidative stress and may aid in the prevention of coronary heart disease, stroke, cancer, and other age-related disorders. In the current study, the flavonoid, vitamin C, total phenolic, β-carotene linoleic acid, and DPPH contents were the most abundant polyphenols in basil grown in soil under a shade-net, all of which act as potent antioxidants and free radical scavengers, stabilizing lipids against peroxidation and inhibiting various types of oxidizing enzymes [38]. There was a stronger relationship found between antioxidant activity and total phenolic content, showing that phenols other than flavonoids are capable of inhibiting free radicals. It can be difficult to quantify how different bioactive compounds contribute to the total antioxidant activity due to their interactions and synergistic effects. Flavonoids contribute to the aroma and flavor of the plant and provide health advantages to humans due to their bioactive capabilities, which include anticancer, anti-aging, anti-inflammatory, antibacterial, and antiviral properties [39].

#### 2.1.5. Condensed Tannins

Condensed tannins are water-soluble antioxidants known as proanthocyanidins, which are extensively dispersed in many plant species as secondary metabolite components and play a significant role in plant defense mechanisms by modifying the resistance of plants to herbivore assault and diseases [40]. Tannins are high molecular weight polyphenolic chemicals that inhibit insects, birds, and herbivores from eating on plants by complexing proteins and imparting an unpleasant taste. Tannins play a significant role in food quality, especially in terms of the key organoleptic qualities that give food its astringent and bitter flavors [41]. Condensed tannins on selected herbs were significantly reduced in a soilless medium and GFT hydroponic system, except for coriander, which had a significantly high amount. In soil cultivation, all herbs had improved condensed tannins with reduced amounts obtained from coriander (Table 1). The low PAR values (418 μmol/m^2^/s) under a non-controlled environment and high day temperature favored the accumulation of tannins. Coriander grown in NTC had the highest condensed tannins (0.18%), which did not differ from levels recorded in the same crop and rocket grown in shade-nets (Table 1). Generally, herbs grown in soilless medium (under NTC) had the least amount of condensed tannins (<0.1). Apart from PAR values, environmental factors, including temperature, water status, photoperiod, soil nutrient content, and genetic factors, contribute to the accumulation of condensed tannin content.

#### 2.1.6. DPPH Antioxidant Capacity

A variety of methods can be used to test the antioxidant capability in various horticultural crops. In the current study, the antioxidant activity of fresh herbs was assessed using the DPPH assay for free radical scavenging ability. When an electron or a free radical species is consumed, DPPH loses its absorption spectra band at 515–528 nm [42]. The DPPH test is a simple, acceptable, and widely used method for assessing plant extracts’ radical scavenging capacity. Antioxidants are plant components that can quench the stable purple-colored DPPH radical to the yellow-colored DPPH radical; natural antioxidants in horticultural crops are attributed to vitamin C, carotenoids, flavonoids, and other phenolic compounds, which are capable of scavenging free radicals and thereby preventing many fatal illnesses [42]. The antioxidant capacity values resulting from DPPH values in the various herb species are investigated (Table 1). The antioxidant capacity of all herbs ranges from 60.27 to 89.74% in the dry samples. The radical scavenging activity of basil and parsley was significantly reduced in the soilless medium under NTC. The maximum antioxidant capacity was found in basil and rocket herbs grown in GFT and in-soil, as well as coriander produced under NTC and parsley grown in-soil. Coriander had the lowest antioxidant capacity, as well as parsley and basil, when grown in a non-controlled plastic tunnel, which can be related to the effects of low PAR. Plant extracts’ antioxidant capabilities are commonly attributed to the presence of phenolic components. Likewise, there is a high positive association between total phenolics, total flavonoids, and vitamin C levels in plants. The antioxidant activity of phenols is substantially determined by their chemical structure. The CHCHCOOH grouping of hydroxycinnamic acids has been discovered to be more capable of proton transfer and radical stabilization than the carboxyl (COOH) grouping of hydroxybenzoic acids [43].

#### 2.1.7. β-Carotene Linoleic Acid Content

The antioxidant activities of phenolic acids were measured by the β-carotene bleaching method carried out in a linoleic acid emulsion system. Phenolic acids can inhibit β-carotene bleaching by neutralizing hydroperoxides and other compounds derived from linoleic acid oxidation [44]. As shown in Table 1, the soil-grown condition did not demonstrate any significant difference between aromatic herbs with respect to β-carotene linoleic acid concentration, but significant differences were observed under GFT and shade-net conditions. The increasing inhibition rate of phenolic acids was observed with the increasing concentrations from 55.2 to 94.2%. The rate of β-carotene bleaching can be slowed down in the presence of antioxidants. Meanwhile, the fennel produced in soil under the shade-net had the highest linoleic acid content (94.2%), significantly inhibited the bleaching of β-carotene, expressing more protective effects although it did not differ significantly (*p* < 0.01) from the content in basil, coriander, parsley, and rocket plants produced in soil under the shade-net as well as the rocket produced GFT under the shade-net. The β-carotene linoleic acid bleaching assay is one of the most widely used methods for determining antioxidants’ ability to inhibit lipid peroxidation during both the initiation and propagation phases. The method works in an aqueous emulsion, generated by the loss of the β-carotene yellow color due to its reaction with radicals, which are formed by linoleic acid oxidation in an emulsion. The herbs grown in soilless medium under an NTC had the lowest inhibition activity of β-carotene linoleic acid. Generally, the herbs grown in soil cultivation had the highest β-carotene linoleic acid concentration compared to herbs grown in the GFT hydroponic system and soilless medium.

### 2.2. Leaf Mineral Content and Yield

Plant foods are endowed with macronutrients such as phosphorus (P), potassium (K), calcium (Ca), magnesium (Mg), and micronutrients such as sodium (Na), copper (Cu), iron (Fe), manganese (Mn), and zinc (Zn). The effect of the mineral content grown in different environmental conditions was determined in this study (Table 2). Although hydroponic structures offer benefits over open-field production, they are never completely resistant to the effects of stressful situations, particularly when it comes to heat and light energy. Additionally, the covering material may alter the greenhouse’s internal light spectrum, which could have an impact on the product’s quality [45]. Overall, the mineral content was greatly influenced by the environment. In terms of the leaf Ca and Mg content, they were highly retained in rocket grown in the soilless medium and GFT, as well as in basil grown in soil. Lower calcium content was observed in other herbs grown in the same environment. Ca deficiency is primarily caused by impaired Ca transportation in the xylem and unstable environmental conditions, which were observed in previous studies on cucumbers [46]. In contrast, rocket had a low content of Zn, Na, and K. The lower mineral element content of the aromatic herbs may be caused by cation competition in the nutrient solution [47]. Coriander had the highest initial ionizable Fe content in all the production systems, while parsley had a moderate level, and basil had the lowest Fe concentration in the soilless medium. Iron is an essential nutrient that participates in a variety of metabolic processes in humans, including DNA synthesis, electron transport, and oxygen transport [47,48]. Parsley also demonstrated increased K and Mn content when grown in a soilless medium and in soil. On the other hand, in the case of the Ca and Mg contents, they were significantly lower in basil, parsley, coriander, and fennel leaves. The leaf content was high in basil grown in soilless medium and in soil, while other herbs showed lower levels of leaf P content in GFT; however, the values were within the recommended range. The recommended daily potassium intake is 3.5 g and can easily be met with diets rich in fruits, vegetables, and herbs. Potassium (K) is needed to maintain electrolyte balance in the body. Potassium deficiency signals are fatigue, irritability, and high blood pressure. Several dried herbs, including coriander, parsley, and basil, have been identified as major sources of K content. Coriander grown in a GFT hydroponic system under a shade net had high leaf Zn content, while rocket had the least. Additionally, Zn is an essential trace element that must be delivered regularly as part of a balanced diet; it participates in immune system regulation, is involved in many cell metabolism processes, and is crucial in sustaining health. Microminerals like Fe, Cu, and Zn are required to produce antioxidant enzymes that engage in the body’s oxidation–reduction reactions. Furthermore, increasing crop leaf fresh mass (yield) is critical for the grower in achieving economic profitability and stability in meeting global food demands, particularly in the current climate scenarios, such as optimizing agricultural practices, managing resources efficiently, and addressing environmental conditions that influence plant growth and development. The study revealed that different production systems had an influence on crop leaf fresh mass. Fresh leaf mass of coriander, parsley, and rocket was not significantly affected by the production system (GFT, in-soil, and in-soilless cultivation). However, basil had high fresh leaf mass when grown in soil, followed by GFT, and it was the lowest in-soilless growth medium. Fennel produced high fresh shoot mass when grown in the GFT system, followed by the in-soil system, although not significantly different from the soilless growth medium. Basil grown in-soil also had increased phytonutrient content observed in plants grown under shade-nets, specifically in terms of vitamin C, total flavonoids, phenolic content, and antioxidant activity in Table 1, which can be attributed to the shade structure’s ability to allow sufficient sunlight penetration for photosynthesis. This regulated light environment supports plant development as well as the buildup of beneficial phytonutritional compounds. The results are in agreement with results found in rocket and other vegetables.

### 2.3. Principal Component Analysis (PCA)

**Figure 1:**
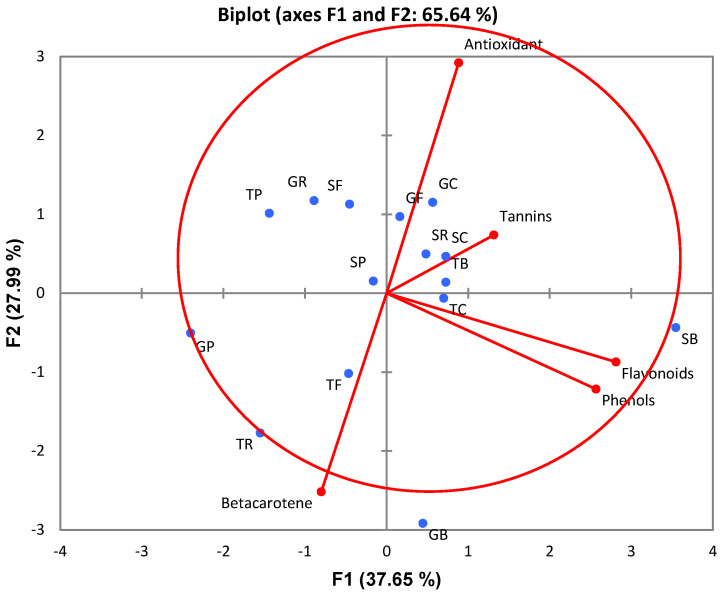
Principal component analysis (PCA) showing correlation loadings of antioxidant constituents in aromatic herbs grown under varied environments (non-controlled temperature tunnel (TP—tunnel parsley, TR—tunnel rocket, TB—Tunnel basil, TF—tunnel fennel, and TC—tunnel coriander) and white shade-nets under both soil (SP—soil parsley, SR—soil rocket, SB—soil basil, SF—soil fennel and soil coriander) and gravel nutrient systems (GP—gravel parsley, GR—gravel rocket, GB—gravel basil, GF—tunnel fennel, and GC—tunnel coriander.

Principal component analysis was carried out to establish a clear relationship between the aromatic herbs grown under varied environments (non-controlled temperature tunnel and white shade-nets) and their variation in phenolic antioxidant constituents. The variance in the data set of quality and bioactive compounds (PC1 37.65% and PC2 27.99%) was explained by the first two principal components or quality parameters. With respect to eigenvalues > 1, two principal components were obtained with their factor loadings. The two PCs explained the x-variables by focusing on five phenolic antioxidant compound parameters that were studied for aromatic herbs in the study, including the antioxidant activity (DPPH (r = 1), beta-carotene (r = −3.54), phenols (r = 0.024), total flavonoids (r = −0.003), and condensed tannins (0.253), as shown in Figure 1. In terms of phytochemicals, herbs responded differentially to the production systems (Table 1). The differences in the phytochemicals evaluated in the study include the total phenolic content, total flavonoids, beta-linoleic acid, and condensed tannin content in all aromatic herbs grown in various production systems. Basil growing in GFT hydroponics and in-soil under a shade-net environment favorably loaded to the PC1 axis was high in terms of the total phenolic content and flavonoids. In contrast, parsley grown in a GFT hydroponic system under shade-net, rocket, and fennel grown in soilless medium under NTC tunnel were negatively loaded in the PC2 axis and displayed strong beta-carotene biosynthesis. At the same time, the environment had shown no effect on the antioxidant activity of the aromatic herbs.

## 3. Materials and Methods

### 3.1. Aromatic Herbs

The experiment was conducted at the Agricultural Research Council (ARC)-Roodeplaat, Pretoria, South Africa (25, 59 S; 28, 35 E and at an altitude of 1200 m above sea level). The five selected herbs in this study were sweet green basil (*Ocimum basilicum* L., Genovese cv.), coriander (*Coriandrum sativum* L., American long cv.), fennel (*Foeniculum vulgare* Mill.), moss curled parsley (*Petroselinum crispum* Mill), and cultivated rocket *(Diplotaxis tenuifolia* L.). Selected herbs were grown in either soil or in a gravel-film technique hydroponic system under a 40% white shade-net and in an open bag hydroponic system using sawdust as a growing medium under a non-temperature-controlled plastic tunnel (NTC). In each production system, five different types of herb seedlings were laid in a randomized complete block design with four replicates.

The herb’s seeds were sown in 200-cavity polystyrene seedling trays filled with commercial seedling medium, Hygromix (Hygrotech South Africa Pty. Limited), and vermiculite was used to cover the seeds after sowing. Seedlings were fertigated with Multifeed^®^ (g/kg) [nitrogen (157 g·kg^−1^), phosphorus (46 g·kg^−1^), potassium (262 g·kg^−1^), sulfur (5.4 g·kg^−1^), magnesium zinc (623 mg·kg^−1^), boron (1004 mg·kg^−1^), ^1^ molybdenum (77 mg·kg^−1^), iron (763 mg·kg^−1^), manganese (205 mg·kg^−1^), and copper (65 mg·kg^−1^)] [AECI Limited, South Africa] at a rate of 1 g L^−1^ of water once daily, from the 2-leaf stage until the transplanting stage. Tinytag T/RH data loggers (Gemini Data Loggers Ltd., West Sussex, UK) were used to record the air temperature (T) and relative humidity (RH) during the experimental trial. The climatic condition during the experimental period in different production systems is shown in Table 3.

#### 3.1.1. Gravel-Film Technique (Closed Hydroponic System)

The description of the gravel film technique (GFT) hydroponic system and the method used to transplant 28-day-old seedlings were followed. Seedlings were transplanted in a 2 m^2^ (1 m × 2 m) plot with a plant spacing of 20 × 20 cm per plot (25 plants/m^2^). The fertigation program described by [49] was followed.

#### 3.1.2. In-Soil Cultivation Under Shade-Net Structure

Five selected herbs were grown in a sandy clay loam soil comprising 68.0% sand, 8.0% silt, and 24.0% clay in the top 30 cm. The chemical composition of the soil consisting of 25.9 mg·kg^−1^ phosphorus (P), 267 mg·kg^−1^ potassium (K), 1313 mg·kg^−1^ calcium (Ca), 437 mg·kg^−1^ magnesium (Mg), and 54.9 mg·kg^−1^ sodium (Na), with a soil pH of 7.5. Twenty-eight-day-old seedlings were transplanted in 2 m × 1 m plots, at a plant spacing of 20 × 20 cm per plot (25 plants/m^2^). Nitrogen was applied in three splits using limestone ammonium nitrate (LAN 28% N) fertilizer as a source, contributing to a total application of 100 kg N/ha. The first application was at planting with 50% N and topdressing twice, every four weeks (25% LAN). All plots were applied with 60 kg P/ha using superphosphate (10.5% P) and 200 kg K/ha using potassium chloride as a basal application at planting.

#### 3.1.3. Soilless Cultivation Under a Non-Temperature-Controlled Plastic Tunnel (NTC)

The NTC (10 m width × 30 m length × 4.2 m height) was covered with a 200-µm light diffusive plastic (Gundle Plastics Pty. Limited, Germiston, South Africa) and relied on natural passive air ventilation to remove heat load in the tunnel by opening flaps and doors on opposite sides.

Twenty-eight-day-old seedlings of herbs were transplanted into 10 L plastic bags (with one seedling planted per bag) filled with sawdust as a growing medium. Herb seedlings were planted in double rows spaced 20 cm and a path-row was 90 cm apart. Under NTC, each block contained 128 plants, and all the herbs were replicated four times. The tunnel carried 782 plants in capacity.

Each plant was irrigated with a dripper delivering 2.1 L/h of nutrient solution. Plants were irrigated with one dripper per plant (at the discharge rate of 35 mL·min^−1^) at 2 h intervals at irrigation cycles of five times daily. The amount of nutrient solution volume was gradually increased as plants enlarged to ensure that 10% to 15% of the applied water leached out of the planting bags, to reduce salt buildup in the growing medium. The locally recommended fertilizers used were Hygroponic^®^ (Hygrotech Pty. Limited, Pretoria, South Africa) comprised nitrogen (N) (68 mg·kg^−1^), phosphorus (42 mg·kg^−1^), potassium (K) (208 mg·kg^−1^), magnesium (30 mg·kg^−1^), sulfur (64 mg·kg^−1^), iron (1.254 mg·kg^−1^), copper (0.022 mg·kg^−1^), zinc (0.149 mg·kg^−1^), manganese (0.299 mg·kg^−1^), boron (0.373 mg·kg^−1^), and molybdenum (0.037 mg·kg^−1^) as well as calcium nitrate [Ca(NO_3_)_2_] comprised N (117 mg·kg^−1^) and calcium (166 mg·kg^−1^). Hydroponic and calcium nitrate fertilizers were applied at 700 g per 1000 L of each, and the water pH of the nutrient solution was maintained at a pH range of 5.5 to 6.5, using nitric acid.

### 3.2. Fresh Leaf Mass

The first harvest was initiated four weeks after transplanting on 10 data plants; thereafter, plants were subjected to leaf harvest at 14-day intervals, which resulted in a total of 6 harvests.

### 3.3. The Phytonutritional Analysis at Harvest

Thirty days after transplanting, the five herb leaves were harvested by cutting and stored in a −80 freezer. Samples were freeze-dried and ground into powder. The auxiliary buds were allowed to regrow after harvesting, and the plants were picked bi-weekly.

#### 3.3.1. Quantification of Vitamin C Content

Leaf extract samples were quantified by weighing 1 g into a test tube and homogenizing it with 10 mL of 5% metaphosphoric acid, following the method described by [50]. The mixture was vortexed and sonicated in an ice-cold water bath for 15 min before being centrifuged at 2000 rpm for 2 min, and samples were filtered using a Whatman No.1 filter paper (Lasec, Cape Town, South Africa). The analysis was then performed on the Prominence-HPLC-PDA model system. Chromatographic separation was achieved using a C18 Luna R column (Shimadzu, Kyoto, Japan), (150 × 4.6 mm, 5 μL) maintained at 25 °C in this experiment; an isocratic mobile phase containing water, acetonitrile, and formic acid (99:0.9:0.1) at a flow rate of 1 mL/min was used in these analyses. The volume of a 20 μL sample was injected, and the detection was set at 245 nm. Sample quantification was achieved based on the calibration curve, and their results were plotted using L-ascorbic acid.

#### 3.3.2. Determination of β-Carotene Content

The β-carotene content was extracted and quantified using the HPLC-PDA method as described by [51] with slight modifications. The herb samples were extracted (0.1 g/mL) with ice-cold hexane: acetone (1:1, *v*/*v*). The extract was vortexed for 2 min before being centrifuged at 2000 rpm for 2 min. The organic phase was transferred and placed on ice in a tube containing a saturated sodium chloride solution. The sample was re-extracted again in an equal amount of time until the extract was colorless. Each time, the extracts were combined with a saturated sodium chloride solution in a test tube. Before injecting the separated organic phase into HPLC, it was filtered through a 0.45 μm syringe filter. The analysis was performed on a Prominence-iHPLC-PDA model system with a sample cooler LC-2030C. (Shimadzu, Kyoto, Japan). A C18 LunaR column (150 × 4.6 mm, 5 μL) kept at 35 °C was used for chromatographic separation. An isocratic mobile phase consisting of acetonitrile: dichloromethane: methanol (7:2:1) was used with a flow rate of 1 mL/min, an injection volume of 20 μL, and detection at 450 nm. Peak identification and quantification were achieved based on an authentic β-carotene standard to plot the calibration curve.

#### 3.3.3. Determination of β-Carotene Linoleic Acid Content

The determination of the percentage of antioxidant activity was assessed by using the β-carotene linoleic acid antioxidant assay following the method described by [52]. The stock solution of β-carotene-linoleic acid was prepared as follows: 10 mg of β-carotene was dissolved in 10 mL of chloroform, 1 mL of solution was added into a tube containing 0.1 mL of linoleic acid and 1 g of Tween 20 and blew by nitrogen to remove chloroform, then 100 mL of distilled water was added into the tube and shaken vigorously. Each sample was prepared in triplicate, and the test systems were incubated in a water bath at 50 °C measured twice at 470 nm before and after 60 min using a FLUOstar-Omega microplate reader (BMG Labtech, Analytik Jena, Jena, Germany) and UV–visible spectrophotometer (Specord 210 plus: Analytik Jena, Jena, Germany). During the incubation of linoleic acid, peroxyl radicals are produced, which are neutralized by the presence of antioxidants; β-carotene bleaching is inhibited, producing a yellow color. Butylated hydroxytoluene (BHT) was used as a positive control. The average β-carotene bleaching rate was calculated at 0 and 60 min, respectively. The antioxidant component (%), which is the overall antioxidant activity of the extract based on the average β-carotene bleaching rate by the extract relative to the aqueous control, was calculated using the following equation:

(1) $$\mathrm{Antioxidant}\;(\%)=\left(\frac{Rcontrol-Rsample}{Rcontrol}\right)\times 100$$

#### 3.3.4. Determination of Condensed Tannins

Tannin contents were determined using an acid-butanol assay according to the method described by [53], using dried samples. Tannins were extracted using 50% methanol and were combined with butanol and hydrochloric acid reagent (95:5 *v*/*v*). Ferric reagent was prepared in 100 mL of 2N HCL with ferric ammonium sulphate and stored in the dark. The samples were then immersed in a 100 °C water bath for an hour before being cooled to room temperature. The amount of condensed tannins in the samples was determined spectrophotometrically at 550 nm (FLUOstar-Omega microplate reader) (BMG Labtech, Ortenberg, Germany). The results were expressed as a percentage of leucocyandin equivalent.

#### 3.3.5. Quantification of the Total Phenolic Content

The total phenolic content of the methanolic herbal extract was determined using the Folin–Ciocalteu reagent described with minor modifications based on [54]. In brief, 250 µL of Folin–Ciocalteu’s reagent and 10 µL of sample were added, followed by 3.5 mL of deionized water. After 3 min, 1 mL of 2% sodium carbonate was added. The mixture was vortexed and incubated at 40 °C for 40 min. It was allowed to cool in the dark. The absorbance was measured at 685 nm using a FLUOstar-Omega microplate reader (BMG Labtech, Ortenberg, Germany). Various gallic acid concentrations were used as the standard for plotting the calibration curve. The total phenolic content results were calculated and expressed in mg gallic acid equivalents (GAE) per g of dry weight (DW).

#### 3.3.6. The Total Flavonoid Content

The total flavonoid content was quantified using the aluminum chloride colorimetric essay method as described by [55] using a FLUOstar-Omega microplate reader (BMG Labtech, Analytik Jena, Jena, Germany). The total flavonoid content (TFC) of the herb extracts was calculated and expressed in mg catechin equivalents (CE) per g of dry weight (DW).

#### 3.3.7. Determination of the Antioxidant Scavenging Activity

The antioxidant activity of the herb extracts’ free radical 2,2-Diphenyl-1-picrylhydrazyl (DPPH) scavenging activity was assessed using the method as described [54], with freshly prepared methanolic DPPH (100 μM). A decline in absorbance by this compound has a linear relationship with the amount of antioxidant activity and was measured after approximately 40 min at 517 nm using a FLUOstar-Omega microplate reader (BMG Labtech, Analytik Jena, Jena, Germany). Ascorbic acid was used as a standard antioxidant. Methanol was used for extraction, served as a blank, and synthetic butylated hydroxy toluene (BHT) was used as a negative control to reduce the error rate. The assay was performed in triplicate. The free radical scavenging activity (RSA) of the vegetable extracts was calculated according to the following formula: RSA (%) = 100 × (1 − AE/AD) where AE is the absorbance of the radical mixture containing the sample extract or standard antioxidant, and AD is the absorbance of the negative control.

#### 3.3.8. Determination of Mineral Nutrients

Dried samples were digested with the nitric-hydrochloric acid according to the procedure described by [56], for the analysis of macro- and micro-nutrients (N, P, K, Ca, Mg, Fe, Cu, Mn, and Zn). An aliquot mixture of the digested solution was boiled gently at 95 °C until the sample had completely dissolved, then filtered through Whatman 42 (2.5-μm particle retention) filter paper. Then, sufficient deionized water was added to bring the final volume up to 50 mL in a volumetric flask. Samples were then analyzed and used for ICP-OES (Inductively Coupled Plasma Optical Emission Spectrometry).

#### 3.3.9. Data Analysis

The phytonutritional compounds and antioxidant activity were determined in triplicate. The data generated were subjected to analysis of variance using the GenStat 64-bit Release 24.1 statistical package. To determine the mean differences, Tukey’s test was used to separate the means. Principal component analysis (PCA) was used to show interrelationships between the variables and detect sample patterns, groupings, and similarities as described using the statistical package STSG Statistica for Windows, version 6.0.

## 4. Conclusions

It is worth noting that aromatic herbs are an easy method to enhance sensory profiles, improve shelf life, and boost phytonutrients in culinary preparations with substantial health benefits. Understanding how the environment influences the accumulation of phytonutritional compounds in plants is critical for long-term efforts to provide healthy food while reducing the environmental impact. The findings reported in the study show that different production systems have a significant impact on yield and the accumulation of health-promoting phytonutritional compounds amongst diverse aromatic herbs. Some have positive ramifications, while others have negative ramifications. Amongst all the five aromatic herbs grown, basil grown in soil under the shade-net had high phytonutrient content compared to other herbs and production systems, except for condensed tannins, which are antinutrient compounds needed in small quantities in the body. The environmental factors under different agronomic practices such as nutrient solution, growing substrates, atmospheric temperature, light quality within shade-nets and tunnels, and radiation may have a significant impact on the production of these phytonutritional compounds and micronutrients in aromatic herbs, with growing substrate and light being the most influential environmental element in the plant tissue. As a result, growers should prioritize in-soil production systems under the shade-net over GFT and NTC to grow aromatic herbs, which have been shown to be easy, sustainable, and environmentally friendly. Finally, this study offered the first foundation for understanding the changes in total phytonutritrional and mineral content in different environments to establish the ideal agronomic experimental conditions that may be regarded as the best system in this research study for consumer health and functional food. It would also be intriguing to explore various abiotic factors on plant growth and their interactions, along with the nutritional content in various growing environments.

## Figures and Tables

**Table 1:** Comparison of the phytonutritional content of basil, coriander, fennel, moss-curled parsley, and rocket leaves on a dry mass basis from different production systems.

Herbs	Phytonutritional Compounds						
	Vitamin C (mg/100 g)	Β-Carotene (mg/100 g)	Total Phenols mg (GAE/g DW)	Total Flavonoids (mg CE/g DW)	Condensed Tannins Leucocyanidin (%)	DPPH Scavenging Activity (%)	B-Carotene Linoleic Acid (%)
**Gravel film technique hydroponic production (shade-net)**							
Basil	13.8 b	36.7 ab	17.1 b	5.4 ab	0.09 de	78.4 b	55.3 h
Coriander	8.3 c	35.9 ab	7.0 de	5.1 abc	0.15 abc	73.6 b	85.5 bcd
Fennel	7.9 c	33.8 abc	8.7 d	5.0 abc	0.06 e	86.9 a	87.9 abc
Parsley	7.9 c	35.3 ab	3.9 h	2.6 d	0.07 e	87.6 a	73.2 ef
Rocket	7.9 c	36.3 ab	7.5 de	3.5 bcd	0.1 cd	86.44 a	88.0 abc
**In-oilless production (Plastic tunnel)**							
Basil	8.3 c	24.6 e	13.2 c	5.3 ab	0.07 e	59.1 c	79.7 de
Coriander	9.2 c	27.6 cde	7.3 de	5.5 ab	0.17 a	89.3 a	69.6 fg
Fennel	8.0 c	25.2 de	8.4 de	3.0 d	0.07 e	89.3 a	75.7 ef
Parsley	8.1 c	25.7 de	5.9 ef	5.0 abc	0.07 e	61.02 c	83.0 cd
Rocket	9.5 c	29.8 bcde	5.9 ef	4.0 bcd	0.07 e	76.9 b	65.8 g
**In-soil production (Shade-net)**							
Basil	18.6 a	39.9 a	25.6 a	6.9 a	0.13 bc	88.6 a	92.3 ab
Coriander	8.0 c	31.5 bcd	9.1 d	5.5 ab	0.12 cd	74.7 b	91.6 ab
Fennel (SB)	7.9 c	32.3 bcd	7.5 de	3.4 bcd	0.14 bc	88.3 a	94.2 a
Parsley (SP)	8.1 c	36.6 ab	7.9 de	4.3 bcd	0.13 bc	87.2 a	90.3 ab
Rocket (SR)	12.4 b	32.7 bc	8.9 d	4.8 abcd	0.15 ab	87.7 a	89.7 abc

Means within each column followed by a different letter are significantly different at the (*p* < 0.001) level.

**Table 2:** Comparison of the mineral composition of basil, coriander, fennel, moss-curled parsley, and rocket leaves on a dry mass basis and yield from different production systems.

Treatment	Leaf Mineral Content (mg/100 g)								Yield (g/Plant)
	Calcium	Iron	Potassium	Magnesium	Manganese	Sodium	Phosphorus	Zinc	
**Gravel film technique production (Shade-net)**									
Basil	1211 bc	10.9 f	2180 fg	339.3 bcd	8.6 bc	68.7 g	717.3 a	4.3 d	122 b
Coriander	651 f	35.7 c	3453 d	322 cd	5.8 de	122 ef	554.7 bc	3.7 ef	67.24 ef
Fennel	1116 bc	35.5 c	3950 bcd	317 cde	6.1 de	463 b	428.7 ef	3.4 fg	78.49 d
Parsley	835 def	77.9 b	5087 a	237 e	11.2 a	151.3 cd	467.3 cdef	4.3 de	51.18 hi
Rocket	1868 a	31.8 cd	3280 de	447 a	3.5 fg	125 e	396.7 f	2.9 g	41.47 ij
**In soilless production (Plastic tunnel)**									
Basil	1267 b	12.9 ef	2440 fg	343.3 bcd	6.7 d	98.6 f	691.3 a	6.2 b	109.6 c
Coriander	745 ef	28.7 cde	3833 cd	326 bcd	6.3 de	133.2 de	528.7 bcd	7 a	70.53 de
Fennel	858 def	14.4 ef	3833 cd	285 de	4.8 ef	469.7 b	431.3 ef	4.6 d	55.9 gh
Parsley	983 def	65.5 b	4433 abc	297.3 de	8.3 c	165 c	480.7 cdef	5.6 c	51.1 hi
Rocket	1894 a	31.6 cd	2630 ef	392 abc	2.5 g	126.3 de	298.5 g	6.5 ab	57.fghi
**In-soil production (Shade-net)**									
Basil	1193 bc	19.5 cdef	2287 fg	405 ab	8.3 c	97.9 f	495.3 bcde	3 g	156.1 a
Coriander	735 f	128.4 a	3407 d	318 cde	8.9 bc	144 cde	583.3 b	3.4 fg	58.7 fgh
Fennel	991 cd	18.3 def	3680 d	311.3 cde	5.2 de	567.3 a	458 def	3 g	62.4 efg
Parsley	1023 cd	119.4 b	4610 ab	294.7 de	10 ab	167.2 c	509.3 bcde	4.5 d	36.3 j
Rocket	1109 bc	61.5 cd	1853 g	307.3 de	3 g	130.1 de	230.1 g	2.1 h	49.1 hi

Means within each column followed by a different letter are significantly different at (*p* < 0.01) level.

**Table 3:** Climatic conditions during the experimental period in a non-temperature-controlled tunnel and a 40% white shade-net.

Microclimate	Temperature (°C)		Relative Humidity (%)	Light Quality
	Maximum	Minimum		PAR (400–700 nm) (μmol m^−2^ s^−1^)
Non-temperature-controlled tunnel	36.5	14	66.9	418
40% White shade-net structure	33.4	13	63.7	683

Temperature non-controlled plastic tunnel (Plastic tunnel), White shade-net (Shade-net). R—red (600–700 nm); FR—far red (700–800 nm); R/FR—red/far red ratio; B—blue (400–500 nm); B/FR ratio; relative transmission of Photosynthetic Active Radiation (PAR).

## Data Availability

Data are contained within the article.
